# Case Report: *MAP2K1 K57N* mutation is associated with primary resistance to anti-EGFR monoclonal antibodies in metastatic colorectal cancer

**DOI:** 10.3389/fonc.2022.1030232

**Published:** 2022-11-07

**Authors:** Gianluca Mauri, Giorgio Patelli, Viviana Gori, Calogero Lauricella, Benedetta Mussolin, Alessio Amatu, Katia Bencardino, Federica Tosi, Erica Bonazzina, Emanuela Bonoldi, Alberto Bardelli, Salvatore Siena, Andrea Sartore-Bianchi

**Affiliations:** ^1^ Department of Oncology and Hemato-Oncology, Università degli Studi di Milano, Milan, Italy; ^2^ IFOM, Istituto Fondazione di Oncologia Molecolare ETS, Milan, Italy; ^3^ Department of Hematology, Oncology, and Molecular Medicine, Grande Ospedale Metropolitano Niguarda, Milan, Italy; ^4^ Candiolo Cancer Institute, FPO-IRCCS, Candiolo, Torino, Italy; ^5^ Department of Oncology, Università degli Studi di Torino, Turin, Italy

**Keywords:** *MAP2K1*, MEK, panitumumab, resistance mechanisms, colorectal cancer

## Abstract

**Background:**

We aim to identify the prevalence and the role of the *MAP2K1 K57N* mutation in predicting resistance to anti-EGFR agents in metastatic colorectal cancer (mCRC) patients.

**Methods:**

We retrospectively reviewed tumor-based next generation sequencing (NGS) results from mCRC patients screened for enrollment in the GO40872/STARTRK-2 clinical trial between July 2019 and March 2021. Then, in patients harboring microsatellite stable (MSS) *RAS* and *BRAF* wild-type *MAP2K1* mutant mCRC, we reviewed outcome to treatment with anti-EGFR monoclonal antibodies.

**Results:**

A total of 246 mCRC patients were screened. Most of them, 215/220 (97.7%), were diagnosed with MSS mCRC and 112/215 (52.1%) with MSS, *RAS* and *BRAF* wild-type mCRC. Among the latter, 2/112 (1.8%) had *MAP2K1 K57N* mutant mCRC and both received anti-EGFR monotherapy as third line treatment. In both patients, *MAP2K1 K57N* mutant tumors proved primary resistant to anti-EGFR agent panitumumab monotherapy. Of interest, one of these patients was treated with anti-EGFR agents three times throughout his course of treatment, achieving some clinical benefit only when associated with other cytotoxic agents (FOLFOX or irinotecan).

**Conclusion:**

We verified in a clinical real-world setting that *MAP2K1 K57N* mutation is a resistance mechanism to anti-EGFR agents in mCRC. Thus, we suggest avoiding the administration of these drugs to MSS *RAS* and *BRAF* wild-type *MAP2K1* N57K mutant mCRC.

## Introduction

Anti-epidermal growth factor receptor (EGFR) monoclonal antibodies (MoAbs) are recommended as standard treatment options for patients affected with *RAS* wild-type (WT), mainly left-sided, metastatic colorectal cancer (mCRC) (1). Anti-EGFR MoAbs prevent EGFR activation, thus blocking the downstream mitogen-activated protein kinase (MAPK) pathway and the resulting proliferative signal (2). Mutations occurring in *KRAS* and *NRAS* exons 2, 3, and 4 represent the main mechanisms of resistance to these anticancer agents, countering the MAPK pathway blockade and even entailing detrimental survival in *RAS* mutant mCRC patients receiving anti-EGFR drugs (3). Accumulating evidence also suggests *BRAF* mutations being a resistance mechanism to anti-EGFR MoAbs, even though treatment is allowed as per label (4). Over the years, several other molecular biomarkers beyond *RAS* and *BRAF* have been associated to primary and/or acquired resistance to anti-EGFR MoAbs, such as gene mutations of *ERBB2*, *EGFR*, *FGFR1*, *PDGFRA*, *PIK3CA*, *PTEN*, *AKT1*, and *MAP2K1*/*MEK1*, amplifications of *KRAS*, *ERBB2*, and *MET*, and fusions of *ALK*, *ROS1*, *NTRK1-3*, and *RET* (5–11). However, given the low prevalence of such alterations and the confounding effect of cytotoxic agents administered together with anti-EGFR MoAbs, no definitive consensus on therapeutic implications has been derived so far.

The increasing availability of next generation sequencing (NGS) panels in the clinical setting allows frequent detection of these alterations, thus requiring evaluation of whether available evidence is strong enough to preclude patients from a potential effective treatment option, therefore posing an emergent therapeutic challenge. Here we provide a molecular tumor board (MTB) discussion of two emblematic cases of uncommon *MAP2K1 K57N* mutated mCRC patients, commenting upon treatment decision making focused on anti-EGFR drug administration.

## Methods

At Niguarda Cancer Center, we retrospectively reviewed tumor-based NGS results of mCRC patients screened for enrollment in the GO40872/STARTRK-2 clinical trial (NCT02568267) between July 2019 and March 2021. Through this process, we checked for mCRC samples harboring *MAP2K1* mutations. After the identification of microsatellite stable (MSS), *RAS* and *BRAF* WT, *MAP2K1* mutant mCRC, we retrospectively reviewed patients’ records to evaluate treatment outcome to anti-EGFR MoAb.

The molecular results presented in this manuscript are based on FoundationOne CDx NGS (Foundation Medicine, Inc.) panel results performed on archival formalin-fixed paraphing-embedded (FFPE) tumor tissue; data from NGS and allele frequency were made available by the sponsor of the GO40872/STARTRK-2 trial upon personalized request, following the approval by the local ethical committee. Concerning mismatch repair (MMR) assessment, results from FoundationOne CDx NGS panels were integrated by immunohistochemistry (IHC) testing performed on archival FFPE tumor tissue, if the NGS data was not available.

After *MAP2K1* mutant mCRC patients’ identification, previously collected plasma samples were retrospectively analyzed by looking for *MAP2K1 K57N* circulating tumor DNA (ctDNA). The ctDNA analysis was performed by droplet-digital PCR (dd-PCR).

Both patients consented to the submission and publication of the following article reports.

## Results

A total of 246 mCRC patients were screened. Most of them, 215/220 (97.7%), were diagnosed with MSS mCRC and 112/215 (52.1%) with MSS, *RAS* and *BRAF* WT mCRC. Among the latter, 2/112 (1.8%) had *MAP2K1 K57N* mutant mCRC and both received anti-EGFR monotherapy as third line treatment.

### Case #1

**Figure 1:**
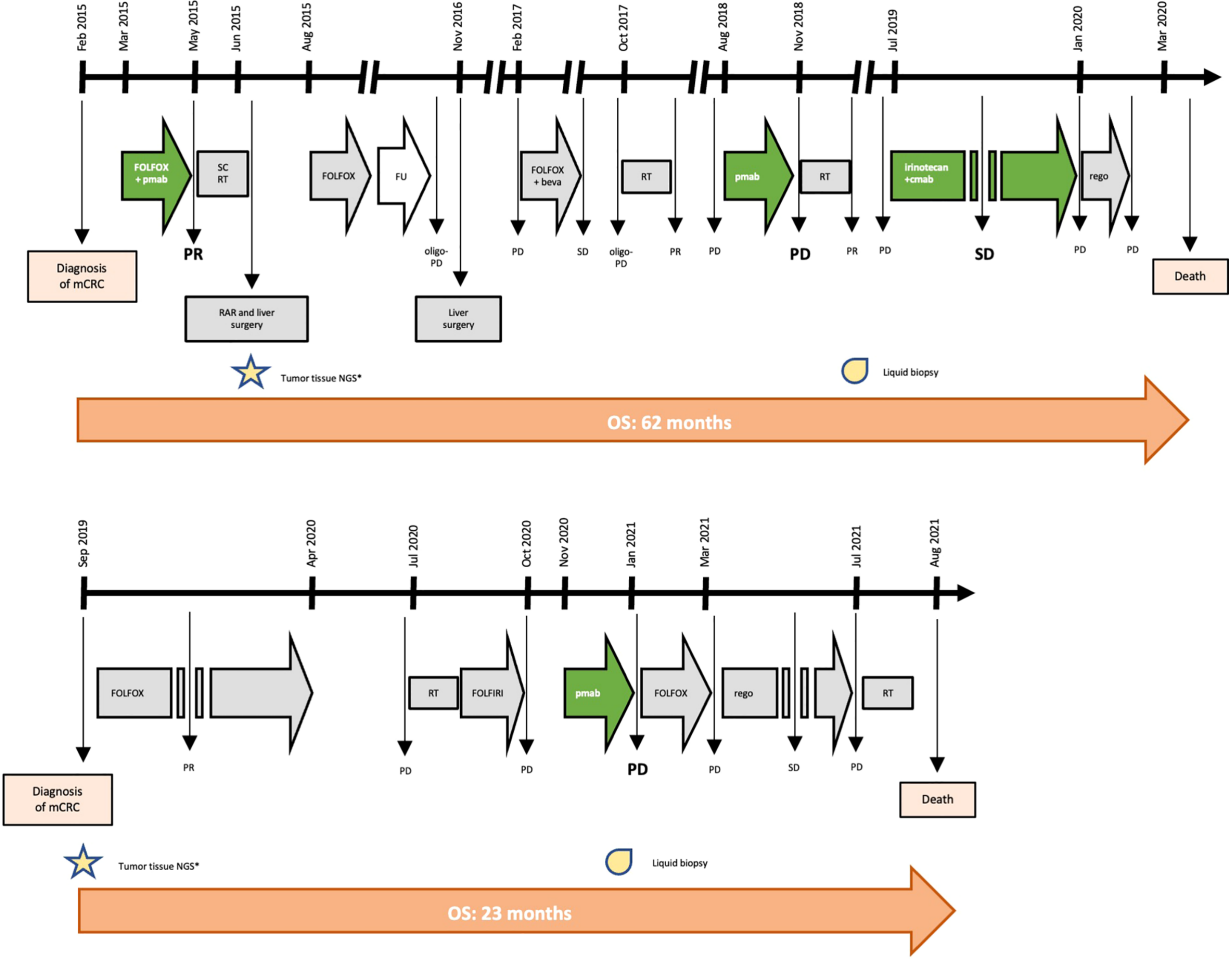
Timeline of treatment for patient #1 and #2. Keys: beva, bevacizumab; cmab, cetuximab; FU, follow-up; mCRC, metastatic colorectal cancer; NGS, next genome sequencing; OS, overall survival; pmab, panitumumab; PD, progressive disease; PR, partial response; RAR, rectal anterior resection; rego, regorafenib; RT, radiotherapy; SC, short-course; SD, stable disease.

In February 2015, a 41-year-old man was diagnosed with stage IV, *RAS* and *BRAF* WT, *ERBB2* not-amplified, MSS rectal cancer with synchronous liver metastases. As presented in **Figure 1** and **Table 1**, the patient underwent multimodal treatment with initial first-line therapy with FOLFOX and panitumumab, achieving partial response (PR) after 4 cycles, followed by short-course radiotherapy (RT) on the pelvis and radical surgery on both the primary tumor and liver metastases in June 2015. Thereafter, the patient received post-surgical treatment with FOLFOX for further 8 cycles. After 11 months of follow-up, a liver recurrence occurred and the patient underwent a second metastasectomy in November 2016. However, due to nodal relapse soon after surgery, FOLFOX and bevacizumab was then reintroduced as second line therapy, achieving stable disease (SD) as best response. Treatment had to be interrupted after 6 courses due to persistent G4 thrombocytopenia, that was interpreted as secondary to 5-fluorouracil infusion. Due to oligo-progressive disease (PD) in a single lymph node, the patient underwent stereotactic RT in October 2017, with no further PD until July 2018. Then, the patient received panitumumab monotherapy as a reintroduction strategy with PD after 6 cycles, developing a severe mediastinal syndrome requiring palliative thoracic surgery and RT. At this stage, aiming to extent the spectrum of druggable therapeutic targets, the patient was screened for the GO40872/STARTRK-2 clinical trial (NCT02568267) at Niguarda Cancer Center, Milan, Italy. Thus, a FoundationOne CDx NGS assay (https://www.foundationmedicine.com/test/foundationone-cdx, Foundation Medicine, Inc.) was obtained from the archival formalin-fixed paraffin-embedded (FFPE) tissue derived from the first liver metastasectomy. The case was presented for MTB discussion, revealing no actionable targets. Of note, a somatic tumor *MAP2K1 K57N* mutation was found on tissue with 17.1% variant allelic frequency (VAF), and confirmed also on liquid biopsy (LB) at progression to anti-EGFR therapy, assessed as reported elsewhere (12) through droplet digital polymerase chain reaction (ddPCR), with a 16.9% minor allelic frequency (MAF). Further treatment with weekly irinotecan and cetuximab was administered, achieving SD as best response. Finally, regorafenib was initiated after PD but soon interrupted due to the deterioration of the clinical conditions. The patient died on March 31^th^, 2020.

**Table 1:** Clinic-pathological features and anti-EGFR treatment outcome for patients #1 and #2.

	Patient #1	Patient #2
Gender	Male	Male
Age at diagnosis (years)	41	62
Primary tumor sidedness	Left	Right
Stage at diagnosis (AJCC 8^th^ edition)	IV	IV
*RAS* status	Wild type	Wild type
*BRAF* status	Wild type	Wild type
*ERBB2* status	Not amplified	Non amplified
MMR status	MSS*	MSS*
TMB (mut/Mb)	3.78	3.78
*MAP2K1* mutation	K57N	K57N
Tumor-tissue NGS		
* Known pathogenic*	*PIK3CA* N345K; *MAP2K1* K57N; *TP53* splice site 375G>A	*APC* R216*; *MAP2K1* K57N; *MYC* amplification; *TP53* T155I
* VUS*	*STAT3* V231M; *ROS1* L686F; *FGFR1* C381Y; *ATRX* I600V; *MAP3K1* S939C; *PDGFRA* T200S; *RAD54L* V119I	*ARFRP1* amplification; *ASXL1* amplification; *AURKA* amplification; *BCL2L1* amplification; *ERCC4* H137D; *GNAS* amplification; *LYN* amplification; *NBN* amplification; *NOTCH1* G2243S; *P2RY8* P9L; *RAD21* amplification; *SRC* amplification; *ZNF217* amplification
Tissue biopsy VAF for *MAP2K1 K57N*	17.1%	14.7%
Liquid biopsy MAF for *MAP2K1 K57N* at progression to anti-EGFR monotherapy	16.9%	0.51%
Best response to anti-EGFR if administered together with other cytotoxic agents (regimen)	PR (FOLFOX + panitumumab)	NA
Best response to anti-EGFR monotherapy (regimen)	PD (panitumumab)	PD (panitumumab)
Progression-free survival with anti-EGFR monotherapy (months)	2.7	1.1
Overall survival (months)	62	23

*, data assessed both by next generation sequencing and immunohistochemistry; MAF, minimal allele frequency; MMR, mismatch repair; MSS, microsatellite stable; NA, never administered; NGS, next generation sequencing; PD, progressive disease; PR, partial response; SD, stable disease; VAF, variant allele frequency; VUS, variant of unknown significance.

### Case #2

In September 2019, a 62-year-old man was diagnosed with *RAS* and *BRAF* WT, *ERBB2* not-amplified, MSS, right-sided mCRC with synchronous liver and bone metastases. Initially, the patient underwent FOLFOX first-line therapy for 12 cycles achieving PR, followed by a therapeutic holiday due to an occurring, non-treatment related intracranial hemorrhagic event. Following systemic PD, the patient received palliative bone RT and FOLFIRI as second line therapy, with further PD. Given the absence of available clinical trials and according to the molecular status, the patient started third line panitumumab monotherapy in October 2020. After 3 cycles, due to worsening clinical conditions, we performed a CT scan demonstrating dimensional and numerical PD. At this stage, aiming to expand druggable therapeutic targets, a FoundationOne CDx NGS panel was performed on archival FFPE tissue from the primary tumor biopsy undergone at baseline. The case was presented at MTB: although no druggable targets were retrieved, a tumor somatic *MAP2K1* N57K mutation was found, with a 14.7% VAF, thus potentially entailing anti-EGFR resistance. This was confirmed by LB through ddPCR showing a 0.51% MAF. Subsequently, the patient was treated with FOLFOX reintroduction achieving PD as best response, and then regorafenib with SD. In July 2021, further palliative RT was provided for worsening gonalgia secondary to a knee bone metastasis. However, the patient died on August 20^th^ 2021 (**Figure 1** and **Table 1**).

## Discussion

We present two cases of patients affected by *RAS* and *BRAF* WT, MSS mCRC, both harboring a *MAP2K1 K57N* mutation, and both treated with anti-EGFR monotherapy with early symptomatic PD. Particularly, patient #1 achieved clinical benefit from anti-EGFR agents only when administered in combination with cytotoxic agents (FOLFOX or irinotecan, respectively), while he rapidly progressed to anti-EGFR monotherapy (**Table 1** and **Figure 1**). Similarly, patient #2 had a right-sided mCRC that proved primary resistant to panitumumab monotherapy as third line of treatment. Altogether, overall survival was consistent with tumor sidedness and other molecular characteristics for both patients.

The presence of a *MAP2K1* mutation was the only anti-EGFR resistance identified in both cases presented; indeed, no other MAPK pathway alterations known as putative mechanisms of resistance to anti-EGFR drugs were found (**Table 1**). For patient #2, we can allege that *MAP2K1* activation exerted a primary mechanism of resistance, as the NGS analysis preceded the administration of the first anti-EGFR therapy. Following, LB confirmed the persistence of this alteration also at the time of PD. Differently, it was not possible to distinguish whether *MAP2K1 K57N* was primary or acquired in patient #1, since archival tissue from initial tissue biopsy was insufficient for NGS analysis; indeed, in this case the NGS analysis was performed on the surgical specimen collected after an initial anti-EGFR based therapy (FOLFOX plus panitumumab). Despite this limitation, we can speculate that it is unlikely that the *MAP2K1 K57N* mutation in patient #1 was acquired upon anti-EGFR exposure, as it has been reported that acquired MAPK pathway alterations are uncommon [6.8% of mCRC patients progressing to first line anti-EGFR containing regimens (13)] and that patient #1 received only 4 courses of FOLFOX and panitumumab (thus having a time-limited anti-EGFR exposure for resistance acquisition).

**Figure 2:**
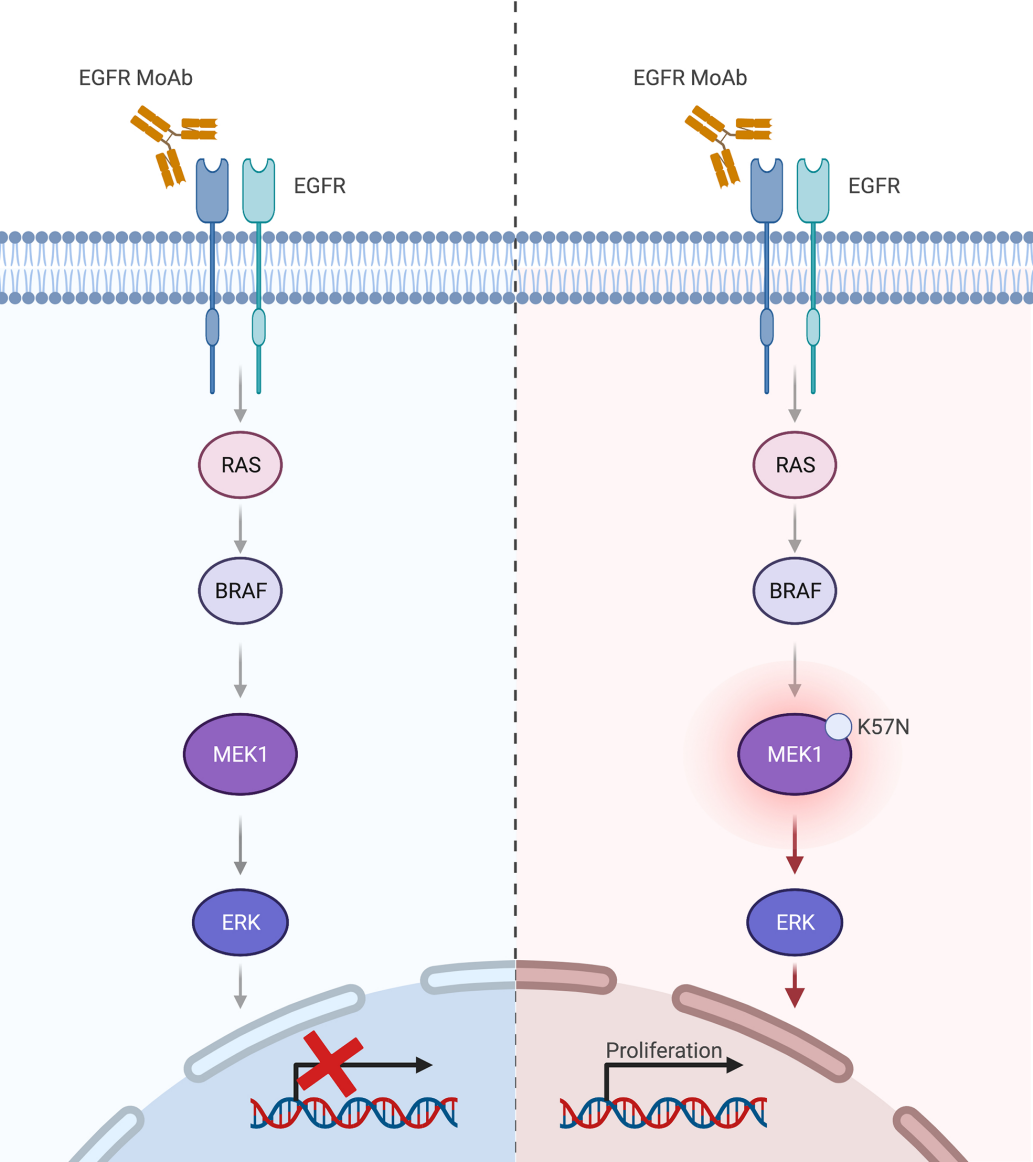
*MAP2K1* K57N in the MAPK pathway drives resistance to anti-EGFR therapy in metastatic colorectal cancer. Keys: MoAb, monoclonal antibodies. Created with BioRender.com.

Notwithstanding, these data provide retrospective evidence that *MAP2K1 K57N* mutation is a potential mechanism of resistance to anti-EGFR MoAbs in mCRC patients. Hence, despite the lack of high level of evidence, *MAP2K1* alterations should be taken into consideration in clinical practice and reviewed through MTB discussion. Indeed, the *Mitogen-Activated Protein Kinase Kinase 1* gene, also known as *MAP2K1*, *MAPKK1* or *MEK1*, is a well-known oncogene encoding for the protein kinase MEK, and exerting its function downstream of the *RAS* and *BRAF* proteins in the MAPK signaling cascade, thus conceivably capable of resistance to MAPK pharmacologic silencing (**Figure 2**) (14). The prevalence of *MAP2K1* mutations in CRC is 1-2%, mainly enriched in *RAS*/*BRAF* WT tumors (https://www.cbioportal.org/). Most common alterations are found in the hotspots K57 (as in both the reported cases) and Q56 codons (15). A previously published mechanism study identified *MAP2K1 K57N* mutations as a class II category mutation, which are partially dependent on upstream *RAF* activation (16). In clinical practice, the role of *MAP2K1* mutations is seldom addressed in mCRC patients, given the absence of specific targeted therapy. However, a contribution to carcinogenesis and resistance to EGFR inhibition has been highlighted in preclinical and translational studies, making *MAP2K1* alterations relevant for MTB discussion before treating these patients with anti-EGFR drugs (at least as monotherapy) (6, 11, 17, 18).

Even if the clinical characterization of patients affected by mCRC harboring this alteration is still partial and not conclusive, comparable cases were previously reported by other authors (15, 19). However, in this prior reports patients with *MAP2K1* mutant tumors were treated with anti-EGFR drugs combined with other cytotoxic or targeted agents, thus hampering definitive clinical conclusions concerning resistance to anti-EGFR MoAbs (15, 19). Besides, only 1 case of *MAP2K1 K57N* was reported in these studies (15, 19). Differently, both our patients were affected by *MAP2K1 K57N* mutant mCRC experiencing PD to panitumumab monotherapy. Given the infrequent occurrence of these alterations, taking into account *MAP2K* mutations in clinical practice and then gathering further clinical evidence in form of scientific reports is needed to translate preclinical discoveries to real-world clinical practice and refine the spectrum of mCRC patients not benefitting from anti-EGFR MoAbs.

In conclusion, we discuss the role of *MAP2K1 K57N* mutation as a negative predictive factor of response and mechanisms of primary resistance to anti-EGFR MoAbs, occurring in 1.8% of *RAS* and *BRAF* WT, MSS mCRC. No assumption can be made on the prognostic value of these alterations given the restricted number of patients, although survival data were in line with expectations in both cases. Therefore, based on previously available literature and the present MTB discussion, anti-EGFR agents should be omitted in *MAP2K1 K57N* mutant mCRC, regardless of primary tumor sidedness, to spare toxicities in patients likely not benefitting from such treatment.

## Data availability statement

The original contributions presented in the study are included in the article/supplementary material. Further inquiries can be directed to the corresponding author.

## Ethics statement

All patients consented to the submission and publication of the following article. This study respected ethical principles as established by the Helsinki Declaration and the Good Clinical Practice: Consolidated Guideline approved by the International Conference on Harmonisation (ICH); a study protocol was presented to the local ethics committee Milano Area 3 (Italy). In no way therapeutic changes took place secondary to the results of this study, in conformity with the aforementioned clinical guidelines for the treatment and management of vulnerable patients. The collection, recording, and reporting of data was accurate and ensured the privacy, health, and welfare of research subjects during and after the study. All collected data will be preserved anonymously and with respect to the patients’ privacy.

## Author contributions

GM, GP, and VG were major contributors in writing the manuscript; CL and EBono performed the pathology procedures that were preparatory to the NGS analysis; BM and AB performed the molecular analyses on plasma samples; GM, GP, VG, AA, KB, FT, EBona, AS-B, and SS provided clinical care to the patients; AS-B and SS supervised oncological care and critically reviewed this article. All authors contributed to the article and approved the submitted version.
