# Corrigendum: Case report: *MAP2K1 K57N* mutation is associated with primary resistance to anti-EGFR monoclonal antibodies in metastatic colorectal cancer

**DOI:** 10.3389/fonc.2023.1147497

**Published:** 2023-03-28

**Authors:** Gianluca Mauri, Giorgio Patelli, Viviana Gori, Calogero Lauricella, Benedetta Mussolin, Alessio Amatu, Katia Bencardino, Federica Tosi, Erica Bonazzina, Emanuela Bonoldi, Alberto Bardelli, Salvatore Siena, Andrea Sartore-Bianchi

**Affiliations:** ^1^ Department of Oncology and Hemato-Oncology, Università degli Studi di Milano, Milan, Italy; ^2^ IFOM, Istituto Fondazione di Oncologia Molecolare ETS, Milan, Italy; ^3^ Department of Hematology, Oncology, and Molecular Medicine, Grande Ospedale Metropolitano Niguarda, Milan, Italy; ^4^ Candiolo Cancer Institute, FPO-IRCCS, Candiolo, Torino, Italy; ^5^ Department of Oncology, Università degli Studi di Torino, Turin, Italy

**Keywords:** *MAP2K1*, MEK, panitumumab, resistance mechanisms, colorectal cancer

**Incorrect Affiliation**

In the published article, there was an error regarding the affiliations for Alberto Bardelli. Affiliation 2 should be removed from Alberto Bardelli. As well as having Affiliation 4, Alberto Bardelli should also have the following new affiliation *Candiolo Cancer Institute, FPO-IRCCS, Candiolo, Torino, Italy*.

There was also an error regarding the affiliation for Benedetta Mussolin. Affiliation 4 should be removed, and the new affiliation [*Candiolo Cancer Institute, FPO-IRCCS, Candiolo, Torino, Italy*] should be added.

The authors apologize for this error and state that this does not change the scientific conclusions of the article in any way. The original article has been updated.

